# Soft tissue vascular tumor-like lesions in adults: imaging and pathological analysis pitfalls per ISSVA classification

**DOI:** 10.1186/s13244-024-01712-w

**Published:** 2024-06-09

**Authors:** C. Marcelin, J. Dubois, V. Kokta, M. F. Giroux, M. A. Danino, S. Mottard, G. Soulez

**Affiliations:** 1 Department of Adult Diagnostic and Interventional Radiology, Hôpital Pellegrin place Amélie-Raba-Léon, 33076 Bordeaux, France; 2 Department of Radiology, Centre Hospitalier de l’Université de Montréal (CHUM), Montréal, QC Canada; 3 Department of Radiology, Radiation Oncology and Nuclear Medecine, Université de Montreal, Montreal, QC Canada; 4 Department of Pediatric Radiology, CHU-Sainte Justine, Montréal, QC Canada; 5 Department of Pathology, CHU-Sainte Justine, Montréal, QC Canada; 6 Department of Surgery, Centre Hospitalier de l’Université de Montréal (CHUM), Montréal, QC Canada; 7 Department of Surgery, Centre Intégré Universitaire de Santé et Services Sociaux (CIUSS) de l’est de L’ile de Montréal, Montréal, QC Canada

**Keywords:** Soft tissue neoplasm, Hemangioma, Vascular malformation, MR imaging, Doppler ultrasound

## Abstract

**Objectives:**

To compare the magnetic resonance imaging (MRI) and Doppler ultrasound (DUS) findings with the pathological findings of soft tissue vascular tumors (STVTs) according to the 2018 ISSVA (International Society for the Study of Vascular Anomalies) classification to differentiate vascular tumors from vascular malformations.

**Methods:**

This retrospective study included patients with STVTs who underwent contrast-enhanced MRI and pathological analysis at our hospital between 2010 and 2020. The presumptive diagnosis based on the on-site imaging and histological analysis was compared with imaging and histological analysis conducted off-site utilizing the ISSVA criteria.

**Results:**

This study included 31 patients with 31 vascular tumors located in the head and neck (*n* = 3), trunk (*n* = 2), and extremities (*n* = 26). The off-site pathological analysis confirmed benign vascular tumors in 54.8% of cases (non-involuting congenital hemangioma: 35.5%; epithelioid hemangioma: 13%; pyogenic granuloma: 3%; and spindle cell hemangioma: 3%). Based on the off-site histological analysis, 25.8% were reclassified as having a vascular malformation whereas three had other benign lesions. Only phleboliths were associated with a vascular malformation (*p* = 0.03). The concordance between off-site MRI and pathological findings was fair (*k* = 0.3902 (0.0531–0.7274)), whereas that between on-site and off-site pathological analyses was poor (*k* = −0.0949 (−0.4661 to 0.2763)).

**Conclusion:**

Benign vascular tumors have non-specific imaging features on imaging with some overlap with atypical vascular malformations. Therefore, histological analysis is recommended. Imaging and pathological analyses should be performed in accordance with the ISSVA classification to minimize inter-observer discrepancies.

**Critical relevance statement:**

Imaging features of benign vascular tumors on MRI are non-specific, leading to discrepancies with pathological findings and potential overlap with atypical vascular malformations. Imaging and histological analyses should be performed in accordance with ISSVA guidelines to improve patient management.

**Key Points:**

- The imaging features of benign vascular tumors are non-specific.
- Histological analysis is recommended for soft tissue vascular tumors in adults.
- Analyses of soft tissue vascular tumors should be performed in accordance with ISSVA guidelines.

**Graphical Abstract:**

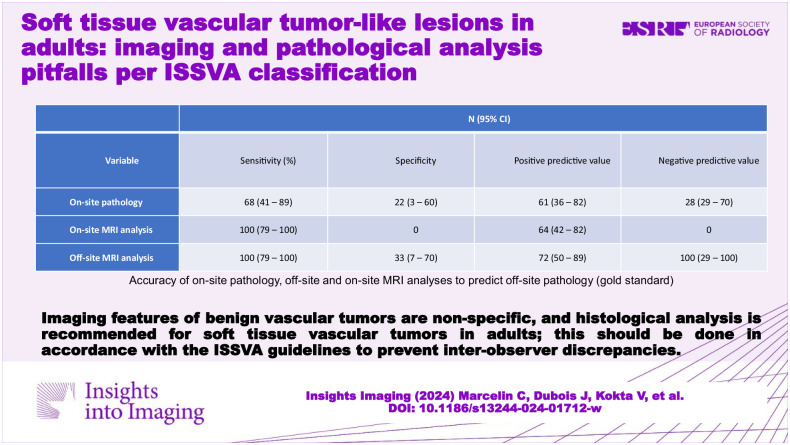

## Introduction

Soft tissue vascular tumors (STVTs) may be present in adults [1, 2]; however, the terminology used to define them is often misleading. In particular, the term hemangioma is often used inappropriately [2]. The 1996 International Society for the Study of Vascular Anomalies (ISSVA) definition is commonly used for vascular tumors and malformations [3] based on the work of Mulliken and Glowacki [4]. The new ISSVA classification for vascular anomalies was adopted in 2014 [5] and revised in 2018 (Table 1). In this classification, vascular tumors are classified as benign, borderline, or malignant. Benign vascular tumors include infantile hemangioma, congenital hemangioma, tufted angioma, spindle cell hemangioma, epithelioid hemangioma, pyogenic granuloma, and rare lesions. Borderline lesions include hemangioendothelioma and Kaposi sarcoma, whereas malignant tumors include angiosarcoma, epithelioid hemangioendothelioma, and rare lesions. Infantile hemangioma (Glut 1-positive) and rapidly involuting congenital hemangioma are not present in adults [6]. Vascular malformation included venous malformations, capillary malformations, lymphatic malformations arteriovenous fistula, and arteriovenous malformations [1].

**Table 1:** ISSVA classification

Vascular tumors	Vascular malformation
*Benign* Infantile hemangioma Rapidly involuting (RICH) Non-involuting (NICH) Partially involuting (PICH) Tufted angioma Spindle-cell hemangioma Epithelioid hemangioma Pyogenic granuloma Others	Capillary malformations Lymphatic malformations Venous malformations Arteriovenous malformations Arteriovenous fistula
*Locally aggressive or borderline* Kaposiform hemangioendothelioma Retiform hemangioendothelioma Papillary intralymphatic angioendothelioma Dabska tumor Composite hemangioendothelioma Pseudomyogenic hemangioendothelioma Polymorphous hemangioendothelioma Hemangioendothelioma not otherwise specified Kaposi sarcoma Others	
*Malignant* Angiosarcoma Epithelioid hemangioendothelioma Others	

Previous studies classified the imaging features of vascular tumors as malignant or benign without applying the ISSVA classification [7–10]. Furthermore, inappropriate terminology was often used, and venous malformations were frequently termed hemangioma or cavernous hemangioma [11].

Most imaging criteria used to differentiate benign and malignant soft tissue masses rely on magnetic resonance imaging (MRI) and do not consider the Doppler ultrasound (DUS) findings, which can be used to characterize congenital hemangiomas [12–15]. Imaging criteria are particularly needed to differentiate benign from borderline and malignant vascular tumors and atypical vascular malformation, in adults. These criteria are needed to select patients requiring biopsy, or surgical excision (for vascular tumor) or percutaneous sclerosis or embolization (for vascular malformation) [2].

The aim of this multicenter study is to compare the MRI and DUS findings with the pathological findings of STVTs according to the 2018 ISSVA classification to differentiate vascular tumors from vascular malformations.

## Materials and methods

### Study design

The institutional review board approved the study protocol for this retrospective chart review and waived the need for obtaining informed consent. Medical records, imaging, and histopathological data from 2010 to 2020 were collected.

Inclusion criteria were patients with a solid tissue mass for > 2 years that was non-compressible on ultrasound and hypervascular on MRI (i.e., predominantly hyperintense on T2-weighted imaging (T2WI), predominantly hypointense on T1-weighted imaging (T1WI), and significant contrast enhancement).

Exclusion criteria were lesions with a typical appearance of a vascular malformation on MRI or DUS, without a soft tissue mass were excluded, children (aged < 18 years) and patients with soft tissue non-vascular tumors, inappropriate MRI technique, and missing histological data.

**Fig. 1:**
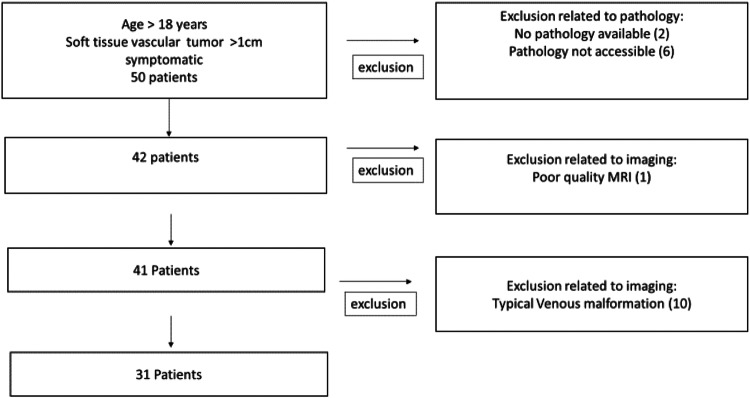
Patient selection flow chart

The onsite radiological, surgical, and pathological databases were searched using the following keywords: soft-tissue tumor, hemangioma, vascular tumor, non-involuting congenital hemangioma, tufted hemangioma, pyogenic granuloma, lobular capillary hemangioma, hemangioma endothelioma, epithelioid hemangioma, angiosarcoma, and Kaposi sarcoma. Figure 1 shows a flow chart of the patient selection process.

We collected the following data: sex, age, signs, and symptoms (pain, bleeding, and compression), presence of congestive heart failure, skin appearance (normal, mass, capillary angioma, and discoloration), number of lesions, age of lesion appearance (birth, puberty, or adulthood), lesion evolution (growth, regression, or stability), lesion location (head and neck, upper or lower limbs, thorax, or abdomen), and clinical and imaging, including DUS and MRI, follow-up findings.

#### MRI

MRI was performed using different 1.5- or 3-Tesla MRI machines after adjustments of coils, field of view, and matrix depending on tumor size, location, and depth. MRI protocols included T1WI before gadolinium chelate administration, T2WI with and without fat suppression (fat saturation and fluid sensitive or short tau inversion recovery sequences), diffusion-weighted imaging (DWI), and T1WI after gadolinium chelate administration with fat suppression (also including the Dixon method or subtraction with pre-contrast imaging).

On-site MRI scans were obtained from the radiology information system and interpreted by three radiologists with expertise in vascular anomalies at one site and by two musculoskeletal radiologists at another site. The diagnosis mentioned on the MRI report was also recorded. Two independent investigators with 8 and 30 years of experience in vascular anomaly imaging, performed off-site reading of the MRI scans. The off-site reading was performed while blinded to the clinical, previous imaging, and pathological information.

The following information was recorded in the off-site reading: the mean anteroposterior and transverse diameters (mm) [16], intramuscular or subcutaneous location, growth pattern defined by the tumor margin (well-defined or irregular, suggesting infiltration of surrounding tissue), tumor circumference (< 25% or ≥ 25%) [17], signal intensity (SI; hypo-, iso-, or hyper-intense compared with adjacent muscle on T1WI, T2WI with fat saturation, and T1WI after gadolinium chelate administration), phleboliths (hypointense round signal voids on T2WI and T1WI), bone invasion (abnormal SI in the cortex or medulla contiguous to the tumor), perilesional edema (hyperintense T2WI), and flow voids (serpiginous or linear structures with low SI on T1WI and T2WI, possibly in communication with feeder vessels). Signal heterogeneity was categorized subjectively as homogenous when < 30% of the lesion was heterogeneous and heterogeneous when ≥ 30% of the lesion was heterogeneous [18].

The SIs of the lesions were objectively evaluated based on comparisons of measurements obtained from the regions of interest covering the largest lesion area with those from similar sized regions of interest in adjacent skeletal muscle and air. Signal-to-noise and contrast-to-noise ratios for the SIs were calculated for each lesion on pre- and post-contrast T1WI. The signal-to-noise ratio was calculated as 0.65 × SI of the region of interest / standard deviation of background noise. The contrast-to-noise ratio was calculated as (the SI of the region of interest – SI of the surrounding tissues) / standard deviation of background noise.

The mean diameter of vascular tumors was calculated manually using the sequence that provided the best contrast with surrounding tissues in the Picture Archiving and Computer System (version 5.2; Impax; Agfa-Gevaert NV, Mortsel, Belgium).

The presumptive diagnosis based on the on-site imaging and histological analysis was compared with imaging and histological analysis conducted off-site utilizing the ISSVA criteria.

#### DUS

The off-site readers obtained the following measurements: longest diameter (mm), margins (well-defined or infiltrative), echogenicity (hypoechoic, anechoic, hyperechoic, or heterogeneous), solid mass (present or absent), fat tissue (hyperechoic mass), calcification (hyperechoic region with acoustic shadowing), vascularity (arterial, venous, or both), shunting (yes or no), venous ectasia (tubular structures with a diameter of more than 1.5 mm with a visible vascular walls) [16], venous lakes (dilated and irregular-shape veins of more than 5 mm in diameter with or without a visible vascular wall) [16], number of vessels (< 2, 2–5, > 5 cm^2^), and resistive index (high [> 0.5] or low [< 0.5]).

#### Histopathological analysis

Independent off-site pathological analysis was performed by a pathologist with 20 years of experience in evaluating vascular anomalies and a pathological diagnosis and subtype were assigned in accordance to the ISSVA classification. A pathological diagnosis and pathological subtype were assigned in accordance with the ISSVA classification. Tumors were classified as benign, intermediate, or malignant. Hematoxylin and eosin staining were performed, and immunohistochemical analysis was performed using anti-Glut-1, anti-D2-40, anti-CD31, anti-CD34, anti-WT-1, anti-p16, and anti-smooth muscle actin antibodies.

#### Statistical analysis

We calculated the sensitivity, specificity, and accuracy of the MRI features in predicting the off-site pathological diagnosis. The association of the MRI features with a vascular tumor or malformation was evaluated using the Mann–Whitney *U* test or Wilcoxon’s non-parametric test for continuous variables, which tests the hypothesis when the median values between the two groups are the same. Categorical variables were analyzed using the chi-squared test if 25% of the cells had a theoretical size of < 5 [unit]; otherwise, Fisher’s exact test was used. The concordances of the on-site and off-site imaging analyses with the off-site histological analysis were evaluated using the Kappa statistic [1].

## Results

### Population

Between 2010 and 2020, 50 consecutive patients with STVTs suspected by MRI or pathological analysis were screened for inclusion in the study. We excluded 19 patients because of incomplete pathological findings, poor imaging quality, or MRI findings not suggestive of STVT (see the patient selection flow chart in Fig. 1). Finally, 31 patients with STVT were included in the study (Table 2). The patients experienced pain (*n* = 31) and symptoms due to compression (*n* = 2). The median delay from on-site MRI to the on-site histological diagnosis was 7 (1–40) months. Pathological analysis was performed on samples obtained from percutaneous biopsy (17/31, 55%) or surgical resection (14/31, 45%).

**Table 2:** Patient characteristics

Age (y) Median (Range)	45.8 (22–79)
Sex	
Men	12 (38%)
Women	19 (62%)
Symptoms	
Pain	31 (100%)
Bleeding	0
Compression	2 (6%)
Congestive heart failure	0
Localization of the lesion	
Head and neck	3 (10%)
Trunk	2 (6%)
Extremities	26 (84%)
Lesion size mm (Range)	43.8 (12–140)
Soft tissue involvement	
Sub-cutaneous	9 (29%)
Intra-muscular	22 (71%)
Histology	
Percutaneous biopsy	17 (55%)
Surgical resection	14 (45%)

The mean clinical and imaging follow-up duration was 49 months (6–108). Four patients were lost to follow-up, two (6%) died, two (6%) developed new symptoms, and eight (25%) had a residual lesion.

### MRI evaluation

We analyzed MRI scans from the 31 patients with 31 vascular tumors located in the head and neck (*n* = 3), trunk (*n* = 2), or extremities (*n* = 26). The mean tumor size was approximately 43.8 (12–140) mm. The MRI findings are summarized in Table 3, as shown only phleboliths were associated with a vascular malformation (*p* = 0.03).

**Table 3:** Association between off-site MRI and pathology analyses

Characteristics	Vascular tumor (*n* = 17)	Vascular malformation (*n* = 10)	*p* value
Sex (women) *n* (%)	11 (69)	5 (55.6)	0.67
Age median (min–max)	58 (22–76)	36 (27–59)	0.034
Fibrous component *n* (%)	13 (81.6)	5 (55.6)	0.2
SNR T1 median (min–max)	12 (3.6; 348)	23 (6; 97)	0.16
CNR T1 median (min–max)	7.8 (−16; 359.5)	14.8 (2; 103.5)	0.57
SNR STIR median (min–max)	29 (3.4; 165)	26.5 (5.7; 55)	0.79
SNR post-contrast median (min–max)	40 (6.7; 433)	23.7 (6.1; 139)	0.28
CNR post contrast median (min–max)	13.4 (−1740; 646)	14.8 (0.8; 121)	0.57
Phlebolith MRI	0	6	0.0365
Peri lesional edema MRI	2	2	0.6016
Fat MRI	3	4	0.6729
Flow void	4	5	0.607

### DUS evaluation

DUS was performed in 15 (48%) of 31 patients. However, statistical analysis of the DUS findings was not possible due to the small sample size. Table 4 presents the DUS and histological features. Interestingly, no calcification was found in patients with vascular malformations on DUS, despite the presence of phleboliths on MRI, likely because we did not specifically evaluate calcifications.

**Table 4:** Doppler ultrasound features and histologic features associated with vasculars tumors and vascular malformations

	Vascular tumor	Vascular malformation
*N* patients with DUS	8	7
Solid mass	8/8 (100%)	7/7 (100%)
Calcification		
Yes	2/8	0/7
No	6/8	7/7
Well defined	4/5	5/5
Infiltration	1/5	0/5
Hypoechoic	3/8	2/7
Hyperechoic	0/8	1/7
Heterogenous	5/8	4/7
Flow		
Arterial	2/8	0/7
Venous	1/8	0/7
Both	5/8	7/7
Shunt		
Yes	2/8	1/7
No	6/8	6/7
Venous ectasia		
Yes	1/8	1/7
No	7/8	6/7
Venous lake		
Yes	0/8	0/7
No	8/8	7//7
Vessel in 2D		
Yes	5/8	2/7
No	3/8	5/7
Number of vessels in cm^2^		
< 2	0/8	0/7
2–5	6/8	5/7
> 5	2/8	2/7
Resistive index		
High	2/3	3/6
Low	1/3	3/6

### Pathological analysis

**Fig. 2:**
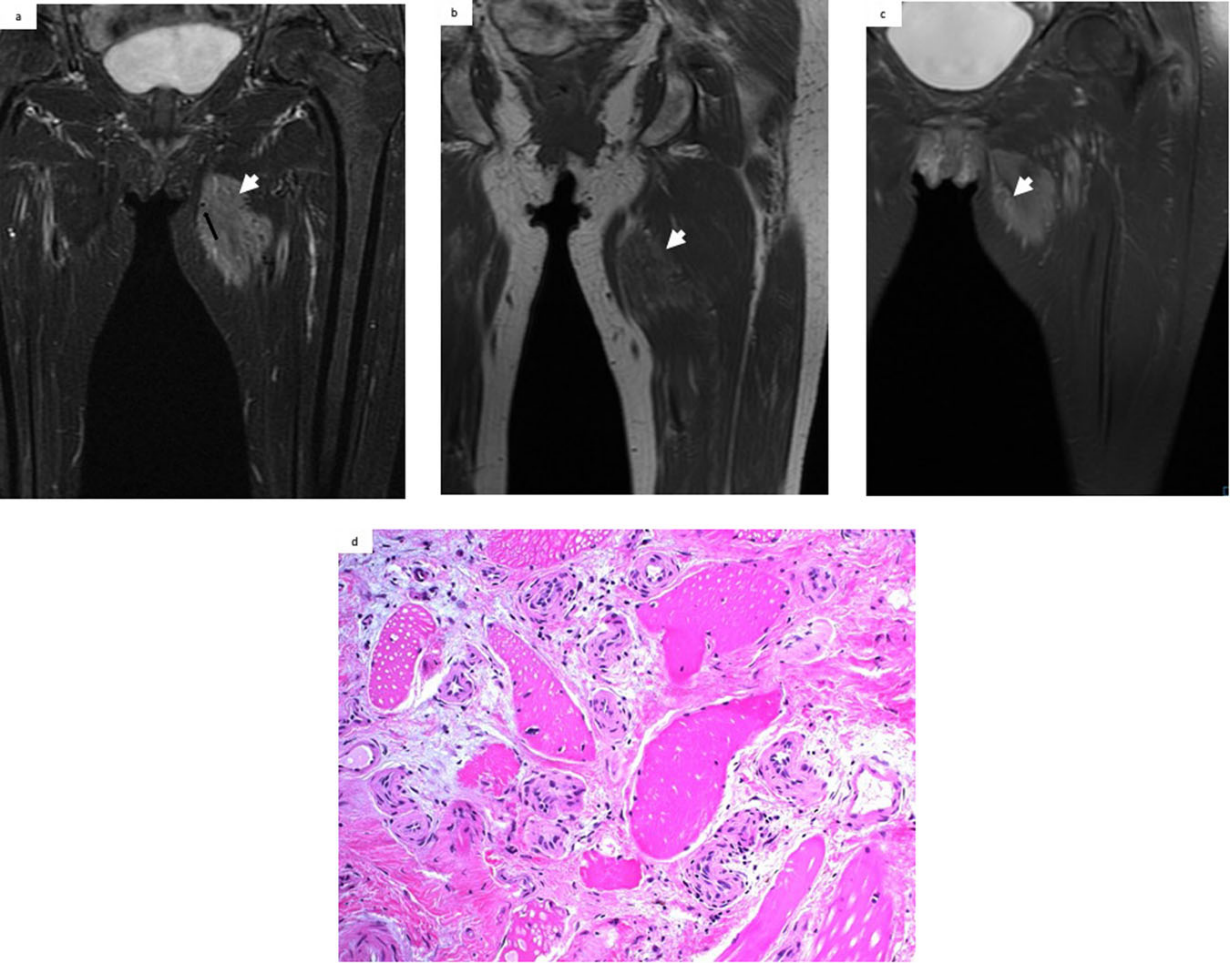
MRI in a 53-year-old man with a painful mass on his left thigh. **a** Heterogeneous hyperintensity appearance of the intramuscular mass (white arrow) on T2WI with fat suppression with flow voids (black arrow). **b** Hypointensity appearance of the intramuscular mass on T1WI (white arrow). **c** Moderate enhancement (white arrow) on delayed contrast-enhanced T1WI with fat suppression. **d** A non-involuting congenital hemangioma (NICH) confirmed by pathological analysis

**Fig. 3:**
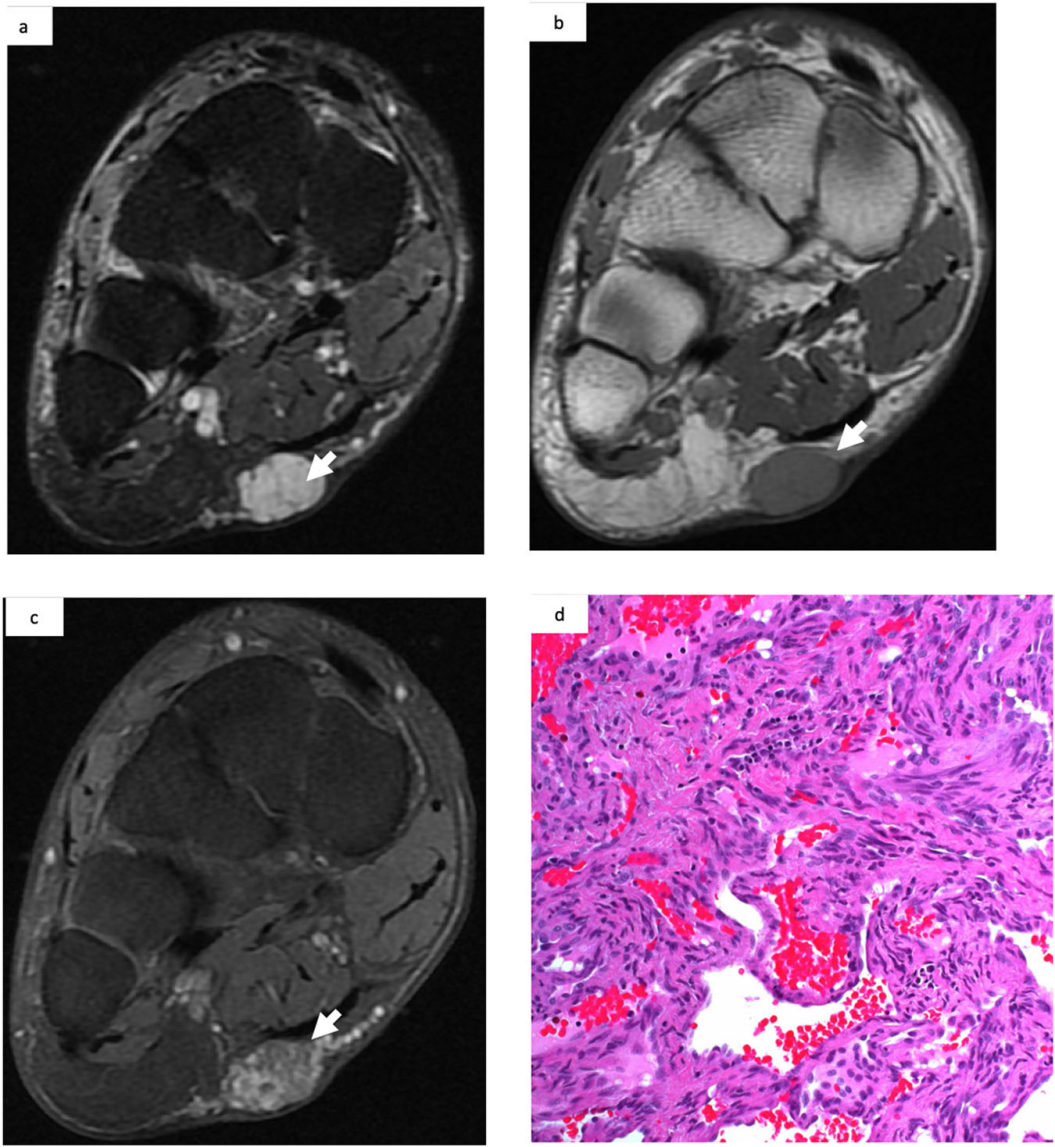
MRI of a 46-year-old woman with a painful mass on her left foot. **a** Hyperintensity appearance of the subcutaneous mass (white arrow) on T2WI with fat suppression. **b** Hypointensity appearance of the mass (white arrow) on T1WI. **c** Patchy enhancement (white arrow) on post-contrast T1WI with fat suppression. Absence of flow voids or presence of phleboliths on different sequences. **d** A spindle cell hemangioma confirmed by pathological analysis

The on-site pathological diagnoses were hemangioma (21/31, 67.7%), vascular malformation (8/31, 25.8%), and other (2/31, 6.5%). In comparison, the off-site pathological diagnoses were benign vascular tumor (17/31, 54.8%), vascular malformation (11/31, 35.6%), and other (3/31, 9.6%). No borderline or malignant vascular tumors were identified. The off-site pathological diagnoses of the confirmed vascular tumors were non-involuting congenital hemangioma (11/31, 35.5%) (Fig. 2), epithelioid hemangioma (4/31, 13%), pyogenic granuloma (1/31, 3%), and spindle cell hemangioma (1/31, 3%) (Fig. 3).

**Fig. 4:**
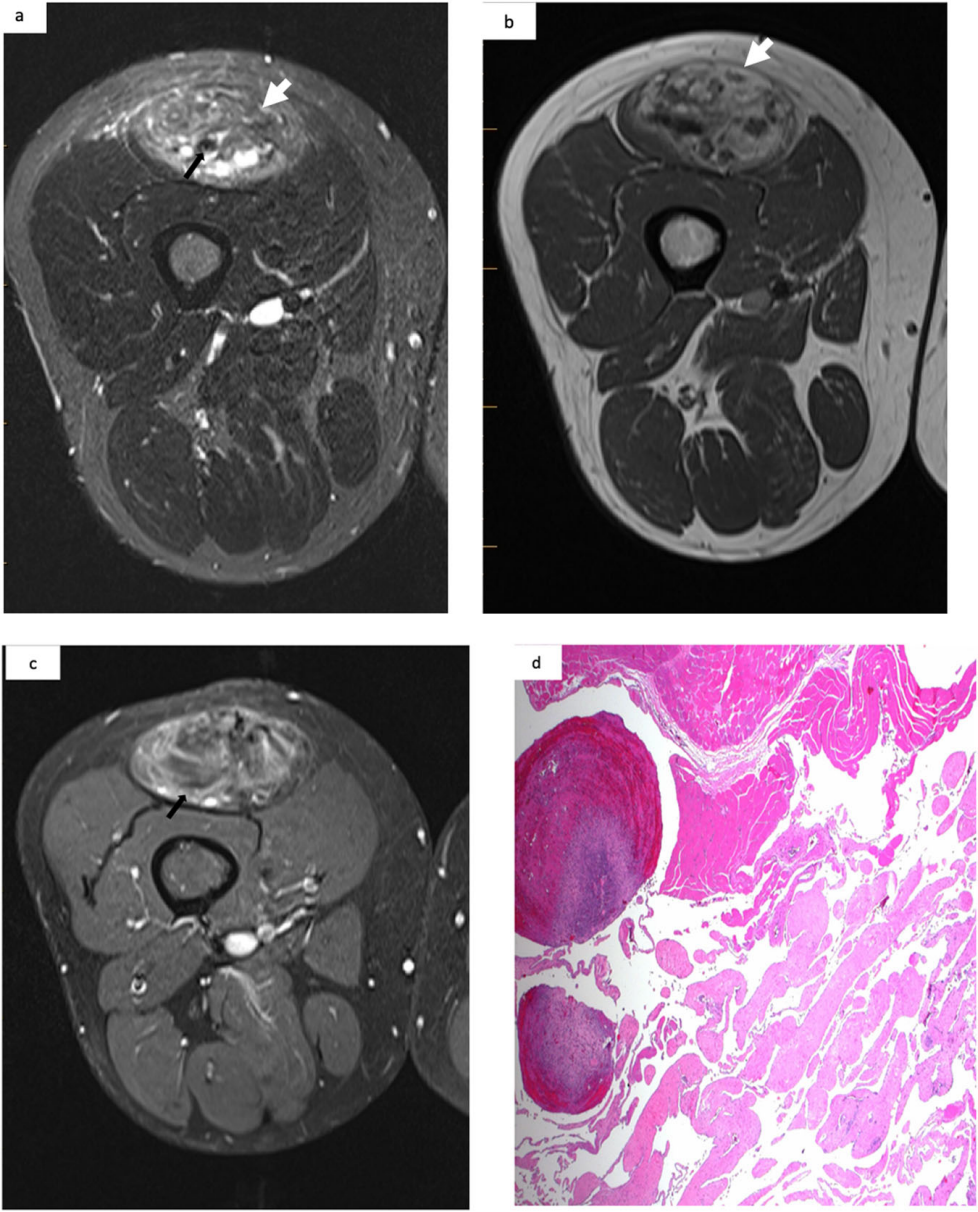
MRI of a 72-year-old woman with a painful mass on her right thigh: **a** Heterogeneous hyperintensity appearance of the intramuscular mass on T2WI with fat suppression (white arrow) with phleboliths (black arrow). **b** Heterogenous hyperintensity appearance of the mass on T1WI (white arrow). **c** Moderate enhancement (black arrow) on delayed contrast-enhanced T1WI with fat suppression. **d** A thrombosed venous malformation indicated by pathological analysis

Eight patients (25.8%) with an on-site pathological diagnosis of vascular tumor were reclassified as vascular malformation following the off-site reading. Three of these patients had thrombosed venous malformations (Fig. 4), two had arteriovenous malformations (AVMs) (however, this diagnosis was incompatible with the MRI and DUS findings), and three had other lesions (9.6%), including myofibroma, leiomyoma, and an undetermined lesion. As shown in Table 3, younger patients were associated with a vascular malformation (*p* = 0.034).

Table S1 (Supplementary Materials) presents the MRI and pathological findings and specimen type used for the pathological analysis.

### Concordance between on and off-site MRI and pathological findings

Table 5 presents the accuracies of the off-site and on-site MRI and the on-site pathological analyses in predicting the off-site pathological findings. The concordance between the off-site imaging and pathological analyses was fair (k = 0.3902; 0.0531–0.7274), indicating fair to moderate agreement [1] between them in differentiating vascular tumors from vascular malformations. The concordance between the on-site and off-site pathological analyses was poor (k = −0.0949; −0.4661 to 0.2763), indicating significant disagreement. Thus, the concordance was better even if far from perfect when radiologist and pathologist use the ISSVA criteria.

**Table 5:** Accuracy of on-site pathology, off-site and on-site MRI analyses to predict off-site pathology (gold standard)

Variable	*N* (95% CI)			
	Sensitivity (%)	Specificity	Positive predictive value	Negative predictive value
On-site pathology	68 (41–89)	22 (3–60)	61 (36–82)	28 (29–70)
On-site MRI analysis	100 (79–100)	0	64 (42–82)	0
Off-site MRI analysis	100 (79–100)	33 (7–70)	72 (50–89)	100 (29–100)

## Discussion

In the present study, MRI & DUS were unable to differentiate between atypical vascular malformations and benign vascular tumors, and biopsy or surgical resection should be performed. In addition, the concordance between expert and non-expert vascular pathologists, as well as that between expert and non-expert vascular radiologists, was poor. There was a significant overlap between STVTs and vascular malformations identified on MRI by experts. A previous study of the imaging patterns of STVTs demonstrated the use of inappropriate terminology [6, 19, 20]. As a result, the ISSVA classification was developed to promote the use of standard terminology [2, 5, 21]. There was fair to moderate concordance between the on-site and off-site imaging analyses. This was mainly because the musculoskeletal radiologists who interpreted the images in the musculoskeletal tumor referral center applied established criteria to exclude malignant soft tissue tumors, and because the vascular anomaly specialist applied the ISSVA classification in the off-site reading. Benign vascular tumors were classified mainly as hemangiomas in the musculoskeletal tumor center.

Liberale et al [11] analyzed 58 studies of head and neck hemangiomas and found that only 14.7% of cases had been diagnosed accurately. After excluding malignant tumors, standard terminology should be used to determine the accurate diagnosis among various vascular anomalies. In the present study, there was a significant overlap between atypical vascular malformations (such as thrombosed venous malformations and STVTs) and other benign tumors that exhibited contrast enhancement.

Imaging is usually reserved for treatment planning as well as for lesions with an unclear diagnosis or deep tissue involvement [21]. MRI using a standardized protocol is the imaging technique of choice to demonstrate the extension and anatomical relationship of the lesion with adjacent structures [22]. T1WI with and without fat saturation, T2WI with fat saturation, and pre- and post-contrast T1WI with fat saturation should be performed to characterize the lesion thoroughly [8]. Unlike AVMs, the MRI protocol for vascular tumors does not routinely include dynamic time-resolved MRI angiography. Delayed contrast-enhanced MRI is preferred for the evaluation of vascular tumors [8]. The use of dynamic time-resolved imaging is recommended for evaluating arteriovenous shunting.

In line with the results of previous studies, we found that a phlebolith was the most reliable indicator of venous malformations, even those that are thrombosed [10, 23, 24]. Typical vascular malformations (venous, lymphatic, and arteriovenous) often have a pathognomonic appearance on MRI and DUS [25–27]. In these cases, biopsy is not required unless somatic genotyping is required to establish the prognosis or initiate targeted medical therapy [28]. Soft tissue edema is not associated with benign tumors, tumor-like lesions, or vascular malformations [26]; and did not have diagnostic value in our study.

Our study had a small sample size; therefore, we could not analyze the role of diffusion restriction on DWI in discriminating vascular tumors from vascular malformations and other benign tumors. DWI has been used to characterize malignant tumors [29], with higher apparent diffusion coefficient values indicating benign lesions [30].

DUS was only performed in half of the patients, mainly those referred to the vascular anomaly specialist. DUS should be performed systematically by radiologists with expertise in vascular anomalies based on relevant criteria for the evaluation of vessel density, resistive index, and shunting [13]. Potential misdiagnosis of AVMs on pathological analysis in one of our cases could have been avoided if the lack of arteriovenous shunting had been found on DUS.

Multidisciplinary management is essential for STVTs. Because MRI and DUS have an insufficient ability to differentiate vascular tumors from atypical vascular malformations and STVTs, a biopsy and/or surgery should be performed for all symptomatic STVTs. The etiopathogenesis of STVTs should be determined on histopathological analysis using the ISSVA classification [31]. Importantly, several pathological findings (AVMs) were not compatible with the imaging findings. Therefore, it is essential to consider the pathological and imaging findings to diagnose AVMs based on the presence of arteriovenous shunting, which is the most important imaging criterion and cannot be assessed on histological analysis.

The differentiation of a benign vascular tumor from a vascular malformation is essential because of differences in their management. Minimally invasive treatments, including percutaneous sclerotherapy [32–34], and percutaneous image-guided thermal ablation, including cryotherapy, are effective for symptomatic venous malformations detected by ultrasound, CT, or MRI and lead to significant decreases in the visual analogue scale (VAS) and volume [35–37].

Unlike infantile hemangiomas, non-involuting congenital, epithelioid, and fusiform hemangiomas do not regress spontaneously [19]. Over a mean follow-up duration of 49 months, 6% of patients developed new symptoms, and 25% had residual vascular lesions on imaging. In cases of residual tumor or symptoms, careful monitoring is required. Surgery is the treatment of choice; recurrence occurs in a small number of patients with a low complication rate [38]. However, incomplete surgical excision is the greatest risk factor for recurrence.

This retrospective series had several limitations. First, there was heterogeneity in the MRI protocol as MRI scans were often acquired outside our hospitals and were reviewed by the study investigators. The fat-suppressed T2WI and fat-suppressed post-contrast T1WI protocols were not standardized. Second, our results may have been affected by recruitment bias as data were collected from vascular and sarcoma clinics. Third, because we searched for patient records using terms related to vascular tumors, the sensitivity of MRI was excellent, but the specificity was poor because other diagnostic entities, such as vascular malformations, were diagnosed as vascular tumors. Fourth, although quantitative imaging features on DWI or dynamic contrast-enhanced MRI may improve the characterization of STVTs, these procedures were not performed. Fifth, off-site analysis of the DUS results was suboptimal because the relevant criteria for diagnosis (e.g., blood flow and calcification) may not have been applied by the radiologist or sonographer. Sixth, we did not analyze the images of non-vascular and malignant vascular tumors, as they were outside the scope of this study.

The two study centers had different pattern of reference, one site being a reference center for soft -tissue tumor with an oncology orientation whereas the other was a vascular anomaly reference center. This can explain the poor concordance between on-site and off-site readings especially for pathology. Finally, pathological analyses were performed on different specimen types, including needle biopsy and surgically resected samples. Biopsy specimens were obtained from 55% of the lesions and may be prone to sampling bias.

In conclusion, benign vascular tumors have non-specific imaging features on MRI and DUS. Histological confirmation is recommended. Pathological analysis should be performed in accordance with the ISSVA classification and be correlated with the clinical presentation and imaging features. Radiologists and pathologists should be informed to refer to the ISVVA classification for imaging and histological analyses to improve patient management.

### Supplementary information

ELECTRONIC SUPPLEMENTARY MATERIAL

## Data Availability

All data and images are stored at CHUM research center and be available upon demand. Data can be shared upon demand and after covering expenses related to data transfer and de-identification.

## Abbreviations

AVM: Arteriovenous malformation
DUS: Doppler ultrasound
DWI: Diffusion-weighted imaging
ISSVA: International Society for the Study of Vascular Anomalies
MRI: Magnetic resonance imaging
SI: Signal intensity
STVT: Soft tissue vascular tumor
