# Supplementary material for: Soft tissue vascular tumor-like lesions in adults: imaging and pathological analysis pitfalls per ISSVA classification
Source: Insights Imaging. 2024 Jun 9;15:135. doi: 10.1186/s13244-024-01712-w (PMC11162993; doi:10.1186/s13244-024-01712-w)
Supplement: Supplementary file 1 — ELECTRONIC SUPPLEMENTARY MATERIAL [file 13244_2024_1712_MOESM1_ESM.pdf]

Soft tissue vascular tumor-like lesions in adults: Imaging and pathological analysis pitfalls per ISSVA classification

ELECTRONIC SUPPLEMENTARY MATERIAL

Table S1 (Supplementary Materials). On-site and off-site diagnoses based on MRI, pathological analyses, and the sampling method**.**

On site MRI	Of site MRI	On site pathology	Off site pathology	
unknown	Vascular tumor	Vascular tumor	Vascular tumor	Biopsy
Vascular tumor	Vascular tumor	Vascular tumor	Vascular malformation	Surgery
Vascular tumor	Vascular tumor	Vascular malformation	Vascular tumor	Biopsy
Vascular tumor	Vascular tumor	unknown	Vascular malformation	Biopsy
Vascular tumor	unknown	Vascular malformation	Vascular malformation	Surgery
Vascular tumor	Vascular tumor	Vascular malformation	Vascular tumor	Surgery
Vascular tumor	Vascular malformation	Vascular malformation	Vascular malformation	Surgery
Vascular tumor	Vascular malformation	Vascular tumor	Vascular malformation	Biopsy
Vascular tumor	unknown	other	other	Surgery
Vascular tumor	Vascular tumor	Vascular tumor	Vascular tumor	Surgery
Vascular tumor	Vascular tumor	Vascular malformation	Vascular tumor	Biopsy
Vascular tumor	Vascular tumor	Vascular tumor	Vascular tumor	Surgery
Vascular tumor	Vascular tumor	Vascular tumor	Vascular tumor	Biopsy
Vascular tumor	Vascular tumor	Vascular tumor	Vascular tumor	Surgery
Vascular tumor	Vascular tumor	Vascular tumor	Vascular malformation	Biopsy
Vascular tumor	Vascular tumor	Vascular malformation	Vascular tumor	Surgery
Vascular tumor	Vascular tumor	Vascular tumor	Vascular tumor	Surgery
Vascular tumor	Vascular malformation	Vascular tumor	Vascular malformation	Biopsy
Vascular tumor	Vascular tumor	Vascular tumor	Vascular malformation	Biopsy
Vascular tumor	Vascular tumor	Vascular tumor	other	Biopsy
Vascular tumor	Vascular tumor	Vascular tumor	Vascular malformation	Biopsy
Vascular tumor	Vascular tumor	Vascular tumor	Vascular tumor	Surgery
Vascular tumor	unknown	Vascular tumor	Vascular tumor	Biopsy
Vascular tumor	Vascular tumor	Vascular tumor	Vascular tumor	Surgery
Vascular tumor	Vascular tumor	Vascular tumor	Vascular tumor	Biopsy
Vascular tumor	unknown	Vascular tumor	other	Biopsy
Vascular tumor	Vascular tumor	Vascular malformation	Vascular malformation	Biopsy
Vascular tumor	Vascular tumor	Vascular tumor	Vascular tumor	Surgery
Vascular tumor	Vascular tumor	Vascular tumor	Vascular malformation	Biopsy
Vascular tumor	Vascular tumor	Vascular tumor	Vascular tumor	Biopsy
Vascular tumor	Vascular tumor	Vascular malformation	Vascular tumor	Surgery
